# Translation and validation of the Traditional Chinese version of the COmprehensive Score for financial Toxicity-Functional Assessment of Chronic Illness Therapy (Version 2)

**DOI:** 10.1186/s12955-020-01646-z

**Published:** 2021-01-08

**Authors:** Dorothy N. S. Chan, Kai Chow Choi, Marques S. N. Ng, Weijie Xing, Bernard M. H. Law, Pui Shan Ho, Cecilia Au, Mandy Chan, Man Tong, Wai Man Ling, Maggie Chan, Suzanne S. S. Mak, Raymond J. Chan, Winnie K. W. So

**Affiliations:** 1 The Nethersole School of Nursing, Faculty of Medicine, The Chinese University of Hong Kong, 7/F Esther Lee Building, Shatin, the New Territories, Hong Kong China; 2 School of Nursing, Fudan University, Shanghai, China; 3 Department of Clinical Oncology, Tuen Mun Hospital, Hong Kong, China; 4 Department of Clinical Oncology, Pamela Youde Nethersole Eastern Hospital, Hong Kong, China; 5 Department of Clinical Oncology, Prince of Wales Hospital, Hong Kong, China; 6 School of Nursing, Queensland University of Technology, Brisbane, Australia; 7 Princess Alexandra Hospital, Metro South Health, Brisbane, Australia

**Keywords:** Comprehensive score for financial toxicity, Psychometric testing, Validity, Reliability

## Abstract

**Background:**

Cancer patients often experience severe financial distress due to the high cost of their treatment, and strategies are needed to objectively measure this financial distress. The COmprehensive Score for financial Toxicity-Functional Assessment of Chronic Illness Therapy (COST-FACIT) is one instrument used to measure such financial distress. This study aimed to translate the COST-FACIT (Version 2) [COST-FACIT-v2] instrument into traditional Chinese (COST-FACIT-v2 [TC]) and evaluate its psychometric properties.

**Methods:**

The Functional Assessment of Chronic Illness Therapy (FACIT) translation method was adopted. The translated version was reviewed by an expert panel and by 20 cancer patients for content validity and face validity, respectively, and 640 cancer patients, recruited from three oncology departments, completed the translated scale. Its reliability was evaluated in terms of internal consistency and test–retest reliability. Confirmatory factor analysis has been used to evaluate the one- and two-factor structures of the instrument reported in the literature. The convergent validity was examined by the correlation with health-related quality of life (HRQoL) and psychological distress. Known-group validity was examined by the difference in the COST-FACIT-v2 (TC) total mean score between groups with different income levels and frequency of health care service use.

**Results:**

The COST-FACIT-v2 (TC) showed good content and face validity and demonstrated high internal consistency (Cronbach’s alpha, 0.86) and acceptable test–retest reliability (intraclass correlation coefficient, 0.71). Confirmatory factor analysis showed that the one- and two-factor structures of the instrument that have been reported in the literature could not be satisfactorily fitted to the data. Psychological distress correlated significantly with the COST-FACIT-v2 (TC) score (r = 0.47; *p* < 0.001). HRQOL showed a weak to moderate negative correlation with the COST-FACIT-v2 (TC) score (r = − 0.23 to − 0.46; *p* < 0.001). Significant differences were seen among the COST-FACIT-v2 (TC) scores obtained in groups of different income level and frequency of health care service use.

**Conclusions:**

The COST-FACIT-v2 (TC) showed some desirable psychometric properties to support its validity and reliability for assessing cancer patients’ level of financial toxicity.

## Background

Patients with cancer and their caregivers often face serious financial distress due to cancer treatments [1], both from medical costs and from reduced income during their treatment or recovery process [2]. Financial distress is a multidimensional concept. In one review [3], although 28% to 48% of cancer patients reported financial distress in monetary terms, 16% to 73% experienced a subjective burden of this distress. The objective and subjective burdens of this financial distress in the context of cancer have been termed ‘financial toxicity’ [4]. A high level of financial toxicity has been demonstrated to increase patients’ symptom burden [5] and reduce their compliance to therapy, health-related quality of life (HRQoL) and survival rate [2, 6]. Therefore, financial issues in the cancer care setting must be discussed to prevent these negative outcomes. A systematic assessment of financial toxicity may help to initiate such discussions and to identify patients who need financial support [7].

The COmprehensive Score for financial Toxicity (COST-FACIT) was developed by de Souza et al. in 2014 [4] and validated in 2017 [8] as a measure of patients’ financial toxicity. The COST-FACIT measure comprises 11 items: one financial item, two resource items and eight affect items [4]. An additional item was added in the COST-FACIT (Version 2) [COST-FACIT-v2] to reflect overall financial wellbeing. This additional item was not included in the calculation of the summary score in the original validation study [8] or the scoring manual (FACIT.org). The COST-FACIT measure has been revealed to have a one-factor structure and has shown high levels of internal consistency and test–retest reliability in the original English version [8]. The COST-FACIT measure has been translated into 10 languages, including Italian and Simplified Chinese [9]. A similar one-factor structure of the COST-FACIT measure has been identified in the 11-item Italian version [10]. However, a two-factor structure of the COST-FACIT measure has been identified in the 11-item Simplified Chinese version [11]. These differences in the factor structures may be due to variations in how health care systems are funded in different regions and in the role of medical insurance in covering patients’ medical and treatment expenses [12].

Although several studies have examined financial toxicity among cancer patients [6], such data are lacking for the Chinese population. In particular, no suitable instrument was available for such measurement in traditional Chinese. In consideration of the possible culture-sensitive characteristics of HRQoL in the diverse background of cancer patients and survivors, the aims of this study were to translate the COST-FACIT-v2 instrument into traditional Chinese (COST-FACIT-v2 [TC]) and to evaluate its psychometric properties—including content validity, internal consistency and test–retest reliability—and its convergent, construct and known-group validity.

## Methods

### Study design

The study consisted of two phases. In the first phase, the original version of the COST-FACIT-v2 was translated from English into traditional Chinese following the Functional Assessment of Chronic Illness Therapy (FACIT) translation method [13, 14] and tested for content validity. In the second phase, the psychometric properties of the COST-FACIT-v2 (TC), including internal consistency, test–retest reliability and convergent, construct and known-group validity, were examined.

### Phase 1: translation, content and face validation

Before the translation process, approval was sought for our use of the FACIT Measurement System. The recommended translation stages in the FACIT translation method were followed. The English version of COST-FACIT-v2 was first translated independently into traditional Chinese by two bilingual translators. One of these translators had a nursing background and the other had no medical or health-related background. A reconciliation of the two forward translations was provided by a third bilingual translator with a science background. Another translator, a native English-speaker fluent in the target language who had no medical or health-related background, independently performed the back-translation of the reconciled version, and a fifth bilingual translator with nursing background reviewed the back-translated version of the scale and compared it with the original English version to assure consistency of linguistic and cultural equivalence. Each of these steps were recorded in a document and sent to FACIT.org.

A panel of 10 experts who care for cancer patients in clinical and community settings and in academic institutions (an oncologist, nurses, university nursing academics specializing in cancer care and social workers at a cancer patient resources center) was invited to evaluate the items’ relevance to the scale and cultural context. Each item was rated on a four-point scale (1 = not relevant, 2 = somewhat relevant, 3 = relevant, 4 = most relevant). The content validity index (CVI) was computed on the item level and the scale level. The item-CVI (I-CVI) was calculated as the proportion of experts who rated the item 3 or 4, and the scale-CVI (S-CVI) was determined as the mean of the I-CVI scores. I-CVI scores of 0.78 and S-CVI scores of 0.90 were considered acceptable indices [15]. Finally, a cohort of 20 patients who were proficient in both English and traditional Chinese were recruited from the oncology departments of two public hospitals and invited to complete the original version of the COST-FACIT-v2 and the traditional Chinese version of the COST-FACIT-v2 and to evaluate the face validity of the scale. The face validity was evaluated by asking the patients to comment on the item appropriateness and interpretability and the time needed to complete the scale.

### Phase 2: psychometric testing of COST-FACIT-v2 (TC)

#### Sample and setting

The study was conducted from November 2018 to January 2019. Cancer patients who were receiving or had completed treatment were recruited from three outpatient clinics of the oncology departments of Hong Kong public hospitals via convenience sampling. The patients were invited to participate in the study while awaiting follow-up. The sample size was determined to provide sufficient subjects for factor analysis. According to Comrey and Lee [16], a sample size of at least 500 subjects could be regarded as very good for performing factor analysis. This sample size is also adequate to detect a correlation coefficient of as little as 0.125 between the COST-FACIT-v2 (TC) and HRQoL and psychological distress (80% power, 2-sided 5% level of significance). To allow for a non-completion rate of up to 20% for some items of the questionnaire, a total of at least 625 patients was targeted for recruitment from the three hospitals.

The inclusion criteria were (1) age of at least 18 years, (2) a diagnosis of stage I-IV of any cancer type, (3) participation in therapy for at least 2 months at the time of the interview and (4) the ability to read traditional Chinese or communicate in Cantonese. Patients with cognitive impairment or language difficulties were excluded.

#### Measurements

A structured, self-report questionnaire was developed for data collection. The questionnaire comprised five sections: (1) sociodemographic characteristics, (2) disease-related characteristics, (3) psychological distress, (4) HRQoL and (5) financial toxicity. The correlations between financial toxicity, psychological distress and HRQoL were used to evaluate the convergent validity of the COST-FACIT-v2 (TC). It has been hypothesized both that greater financial toxicity would have a mild to moderate negative correlation with HRQoL [8, 17, 18], and that greater financial toxicity would have a mild to moderate positive correlation with psychological distress [8].

A distress thermometer was used to evaluate the patients’ level of psychological distress. The distress thermometer was a self-reported, pencil-and-paper measurement on a scale of 0 to 10, where 0 indicated ‘no distress’ and 10 indicated ‘extreme distress’ [19]. The distress thermometer has been validated for use in Chinese cancer patients and has shown good reliability [20]. The Functional Assessment of Cancer Therapy-General (FACT-G-Version 4) was used to measure HRQoL [21]. The FACT‐G, developed initially in English, has been translated into traditional Chinese and validated for use in Chinese cancer patients [22]. This 27-item instrument has four subscales for physical, social/family, emotional and functional well‐being. Each item was scored on a five-point rating scale (0 = ‘not at all’, 1 = ‘a little bit’, 2 = ‘somewhat’, 3 = ‘quite a bit’ and 4 = ‘very much’). Negative items were scored in reverse before summing the total score. Higher scores on the subscales and on the total score indicated better HRQoL.

The COST-FACIT-v2 (TC) was used to assess the patients’ financial toxicity. Each item was rated on a five-point scale (0 = ‘not at all’, 1 = ‘a little bit’, 2 = ‘somewhat’, 3 = ‘quite a bit’ and 4 = ‘very much’). The financial toxicity score was calculated by summing the 11 items (items 2, 3, 4, 5, 8, 9 and 10 were reverse-scored), multiplying by 11, and dividing by the number of items answered; a lower score indicated greater financial toxicity [4]. In addition, the development of the COST-FACIT-v2 (TC) involved (1) translation, content and face validation and (2) psychometric testing (reliability and validity testing).

#### Data collection

Eligible subjects were approached by the research staff during their regular medical consultation. After informed consent forms were collected, the patients were invited to complete the demographic questionnaire and the COST-FACIT-v2 (TC). The questionnaires were returned to the researcher as soon as they were completed.

#### Data analysis

The normality of variables with continuous data, including total and subscale scores, was assessed based on their skewness statistics and normal probability plots. Minimal deviations from normal distribution were found. The patients’ demographic and disease-related characteristics were summarized and reported in appropriate descriptive statistics, including frequency, percentage, mean and standard deviation (SD). The subscale and total scores of the instruments, including the COST-FACIT-v2 (TC) and FACT-G, were presented via means and SDs. The content validity was calculated for the COST-FACIT-v2 (TC). The internal consistency of the COST-FACIT-v2 (TC) overall scale and of each sub-scale were assessed using Cronbach’s alpha, where an alpha coefficient of 0.7 or greater was considered acceptable. The intraclass correlation coefficient (ICC) was used to evaluate the scale’s test–retest reliability. An ICC of greater than 0.7 was considered acceptable [15].

Confirmatory factor analysis (CFA) was performed using LISREL 8.8 (Scientific Software International, Inc.) to test the one-factor and two-factor structures of the COST-FACIT-v2 (TC) suggested by the original version of de Souza et al. [4] and the simplified Chinese version of Yu et al. [11]. The parameters were estimated by the robust diagonally weighted least-squares method, which allows violation of the multivariate normality assumption of the item data. Because the chi-square test is sensitive to sample size and violation of the multivariate normality assumption [23], several goodness-of-fit indices were used to assess the overall fit of the CFA model. Guided by Schermelleh-Engel et al. [23], the following fit indices were chosen: (1) the Satorra-Bentler scaled chi-square statistic to degree of freedom ratio (χ^2^/*df*), (2) the root-mean-square error of approximation (RMSEA), (3) the standardised root-mean-square residual (SRMR), (4) the adjusted goodness-of-fit index (AGFI), (5) the comparative fit index (CFI) and (6) the non-normed fit index (NNFI), which is also called the Tucker-Lewis index (TLI). An acceptable model fit was indicated by a χ^2^/*df* value of no greater than 3, an SRMR value of 0.1 or less, an RMSEA value of 0.08 or less, an NNFI score of at least 0.95 and an AGFI or CFI value of at least 0.9 [23].

Convergent validity was tested by determining the correlations between the COST-FACIT-v2 (TC), HRQoL and psychological distress, using the Pearson’s correlation coefficient [8, 17, 18]. Known-group validity was evaluated by comparing the average COST-FACIT-v2 (TC) scores among various groups of patients. A previous study demonstrated that individuals who had lower income and higher level of health care use would have higher financial toxicity [8]. Therefore, a *t*-test was used to compare the mean scores of the COST-FACIT-v2 (TC) among patients in various income and health care use groups. All statistical analyses except CFA were performed with IBM SPSS version 24 (IBM Corp., Armonk, NY). All statistical tests were two-sided, and the level of significance was set at 0.05.

### Ethical considerations

Ethical approval for the study was obtained from the Clinical Research and Ethics Committee of the study institution on 2 October 2018. All eligible subjects received an information sheet with details of the study, their rights regarding participation in the study and withdrawal, and data confidentiality. Each patient who was interested in participating in the study signed a consent form and returned it with the completed questionnaire to the clinical site investigator. The study researchers also sought permission to access the patients’ medical records. All information collected about the participants was kept strictly confidential.

## Results

### Phase 1 results

#### Content validity and face validity of COST-FACIT-v2 (TC)

The COST-FACIT-v2 (TC) demonstrated good content validity: the I-CVI of the 12 items ranged from 0.8 to 1.0, and the S-CVI was 0.97. Two experts commented that item 4 (‘I feel I have no choice about the amount of money I spend on care’) would better be written as ‘I feel it is necessary for me to spend the amount of money on care’, and they thought it would be easily understood by patients. However, the suggested amendment would have reversed the original meaning of the item. After further discussion, the original version was retained. The face validity of the COST-FACIT-v2 (TC) established that it could be completed in 15 min. All 20 patients commented that they had no difficulty understanding and answering the questions.

### Phase 2 results

#### Participant characteristics

A total of 640 patients consented to participate in this phase of the study and completed the questionnaire. Their mean age was 59.9 years (SD, 11.1), and around two-thirds were female. Nearly 80% were not employed. Most participants lived with their families. The median time since their first diagnosis of disease was 14 months. More than 70% of them had stage III or IV disease. Around 36% and 25% of the cancer patients had breast cancer or gastric and colorectal cancer, respectively. Surgery, radiotherapy and chemotherapy were the most common treatment types (Table 1).

**Table 1:** Socio-demographic, disease and medical finance characteristics of the study sample (N = 640)

*Socio-demographic characteristics*	
Age (years)^a^	59.9 (11.1)
Sex	
Male	229 (35.8%)
Female	411 (64.2%)
Marital status	
Single/divorced/widowed	179 (28.0%)
Married/cohabitation	461 (72.0%)
Educational level	
No formal education/primary	202 (31.6%)
Secondary	325 (50.8%)
Post-secondary or above	113 (17.7%)
Have full-/part-time job	
No	507 (79.2%)
Yes	133 (20.8%)
Household monthly income (HK$)	
< 10,000	259 (40.5%)
10,000–29,999	197 (30.8%)
≥ 30,000	144 (22.5%)
Don’t know	40 (6.3%)
Living alone	
No	585 (91.4%)
Yes	55 (8.6%)
Time traveling from home to hospital (minutes)^b^	60 (40–90)
*Disease characteristics*	
Time since diagnosis (months)^b^	14 (6–36)
Stage of disease	
I	49 (7.7%)
II	111 (17.3%)
III	172 (26.9%)
IV	303 (47.3%)
Unsure	5 (0.8%)
Specific sites of the cancer	
Breast	233 (36.4%)
Gynecological	27 (4.2%)
Head and neck	34 (5.3%)
Gastric and colorectal	162 (25.3%)
Genitourinary	35 (5.5%)
Lung	104 (16.2%)
Hematological	25 (3.9%)
Skin, bone and soft tissue	10 (1.6%)
Brain and central nervous system, endocrine glands and others	10 (1.6%)
Type of treatment received	
Surgery	417 (65.2%)
Radiotherapy	290 (45.3%)
Chemotherapy	522 (81.6%)
Target therapy	264 (41.3%)
Hormonal therapy	141 (22.0%)
Immunotherapy	19 (3.0%)
Admitted to A&E or hospital due to cancer related complications in the last year	
No	364 (56.9%)
Yes	276 (43.1%)
Admitted to A&E due to cancer related complications in the last year	
0	410 (64.4%)
1–2	169 (26.5%)
≥ 3	58 (9.1%)
Admitted to hospital due to cancer related complications in the last year	
0	384 (60.3%)
1–2	194 (30.5%)
≥ 3	59 (9.3%)
Any co-existing disease	
No	387 (60.5%)
Yes	253 (39.5%)
*Medical finance characteristics*	
Sources of finance for the cancer treatment	
Personal income	120 (18.8%)
Personal savings	496 (77.5%)
Personal medical insurance	164 (25.6%)
Children	194 (30.3%)
Parents	8 (1.3%)
Spouse	111 (17.3%)
Civil service medical and dental benefits	62 (9.7%)
CSSA	35 (5.5%)
Social security allowance scheme	319 (49.8%)
Governmental medical fee waiving mechanism	24 (3.8%)
Other drugs or medical assistance programs	116 (18.1%)
Ever discussed with health professionals about medical fee	
No	239 (37.3%)
Yes	401 (62.7%)
Willing to discuss with health professionals about medical fee	
No	29 (4.5%)
Yes	611 (95.5%)
Have medical Insurance	
No	424 (66.3%)
Yes	216 (33.8%)
*Among those who have medical insurance (n* = *216)*	
Type of medical insurance	
Personal	166 (77.2%)
Company	24 (11.2%)
Both	25 (11.6%)
Insured amount per month (HK$)	
≤ 500	47 (21.8%)
501–1,000	78 (36.1%)
> 1,000	73 (33.8%)
Don’t know	18 (8.3%)

Data marked with ^a^are presented as mean (standard deviation) and with ^b^as median (inter-quartile range), all others are presented as frequency (%)

A&E, Accident and Emergency Department; CSSA, Comprehensive Social Security Assistance

#### Internal consistency and test–retest reliability

The Cronbach’s alpha value for the scale was 0.86, which indicates good internal consistency, and the ICC was 0.714 (95% confidence interval: 0.545–0.827), which indicates acceptable test–retest reliability.

#### Convergent validity

The COST-FACIT-v2 (TC) total score showed significant correlation with the overall scale and with the subscales of HRQoL and psychological distress. The correlation between the COST-FACIT-v2 (TC) and HRQoL ranged between − 0.23 and − 0.42, which indicated a weak-to-moderate negative correlation (weak correlation: r = 0 to − 0.3; moderate correlation: r = − 0.3 to − 0.7; strong correlation: r = − 0.7 to − 1.0) [24] and supported the convergent validity of the scale. The COST-FACIT-v2 (TC) total score also showed a statistically significant moderate positive correlation (0.47) with the distress thermometer (psychological distress) (Table 2).

**Table 2:** Health-related quality of life and financial toxicity measures and their correlations with COST-FACIT-v2 (TC) total score among the participants

	Mean (SD)	Correlation with COST total score
*Health-related quality of life (FACT)*		
Physical well-being (PWB) [range: 0–28]	19.2 (5.8)	− 0.34 (*p* < 0.001)
Social/family well-being (SWB) [range: 0–28]	19.1 (5.1)	− 0.23 (*p* < 0.001)
Emotional well-being (EWB) [range: 0–24]	17.7 (4.4)	− 0.42 (*p* < 0.001)
Functional well-being (FWB) [range: 0–28]	15.7 (5.4)	− 0.39 (*p* < 0.001)
FACT-G total score [range: 0–108]	71.6 (15.5)	− 0.46 (*p* < 0.001)
*Psychological distress*		
Distress thermometer [range:0–10]	6.0 (3.2)	0.47 (*p* < 0.001)

COST-FACIT-v2 (TC), COmprehensive Score for financial Toxicity (Version 2)—traditional Chinese; EWB, emotional well-being; FACT-G, Functional Assessment of Cancer Therapy—General; FWB, functional well-being; PWB, Physical well-being; SWB, social/family well-being

#### Known-group validity

Known-group validity was established based on the differences in the COST-FACIT-v2 (TC) total mean score in different household monthly income groups and health care use groups. The results show that participants with lower monthly household income had higher COST-FACIT-v2 (TC) total mean scores, whereas the reverse was noted in the higher income group. A significant difference in the COST-FACIT-v2 (TC) total mean score was also seen among participants who had been admitted to an Accident and Emergency Department (A&E) or a hospital at least once over the past year. A lower overall COST-FACIT-v2 (TC) mean score was noted in those who had not been admitted to A&E or a hospital over the past year (Table 3).

**Table 3:** Known group comparisons on COST-FACIT-v2 (TC) total score

	COST-FACIT-v2 (TC) total score (Mean [SD])
Household monthly income (HK$)	
< 10,000	25.0 (8.4)
10,000–29,999	24.9 (8.9)
≥ 30,000	20.7 (8.8)
*p* value	< 0.001
Admitted to A&E or hospital due to cancer related complications in the last year	
No	22.5 (8.9)
Yes	25.8 (8.3)
*p* value	< 0.001
Admitted to A&E due to cancer related complications in the last year	
0	22.7 (8.8)
1–2	25.6 (8.1)
≥ 3	27.4 (9.5)
*p* value	< 0.001
Admitted to hospital due to cancer related complications in the last year	
0	22.8 (9.0)
1–2	25.1 (7.7)
≥ 3	27.0 (9.9)
*p* value	< 0.001

A&E, Accident and Emergency Department; COST-FACIT-v2 (TC), COmprehensive Score for financial Toxicity (Version 2)—traditional Chinese

#### Factorial validity

Both the one-factor and two-factor structures of the COST-FACIT that have been reported in the literature [4, 11] were evaluated by CFA. The results of the CFA indicated that neither the one-factor structure (χ^2^ = 639.6, df = 44, *p* < 0.001; RMSEA = 0.146; SRMR = 0.093; CFI = 0.91; NNFI = 0.91; AGFI = 0.96) nor the two-factor structure (χ^2^ = 504.9, df = 43, *p* < 0.001; RMSEA = 0.134; SRMR = 0.092; CFI = 0.93; NNFI = 0.93; AGFI = 0.95) satisfactorily fitted the data.

## Discussion

The findings of this study provide some desirable psychometric evidence to support the validity and reliability of the traditional Chinese version of the COST-FACIT-v2 to measure cancer patients’ financial toxicity. High I-CVI and S-CVI scores were obtained for this instrument, which indicated the relevance of the concept being measured and the socio-cultural relevance of the tool to be used in the local population and setting.

The reliability of the scale was established with a Cronbach’s alpha value of 0.86. This finding was comparable to that of the original version [8]. The obtained value was well above the reference value of 0.7, and the high internal consistency indicated the homogeneity of the scale and its strong reliability in various languages. The test–retest reliability of the 11 items in the scale showed an acceptable ICC in the test and retest period, which reflects the stability of the scale. This value was also comparable to that of the original version [8].

The factorial validity of the COST-FACIT-v2 (TC) was however not yet established by CFA. Both the one-factor structure reported by the original version of de Souza et al. [4] and the two-factor structure of the simplified Chinese version of Yu et al. [11] were not satisfactorily fitted by our data. Additional studies are warranted to examine its factorial structure.

The most common source of financial support for cancer treatment for the patients in this study was their personal savings, followed by funding under the social security allowance scheme, financial support from their children and personal medical insurance. These finance sources seem sufficient for the expenditures from cancer treatment in the short term. However, patients also considered their long-term expenditures because their savings may eventually be depleted, and their medical insurance may not be adequate to cover the required expenditures thereafter. In addition, most of the patients were not employed during the recruitment period, which further exacerbated their concerns over their ability to pay for further treatment and care. To meet the needs of their present treatment and prepare for the future, patients may have to adopt some coping strategies, such as application for additional financial assistance and reducing expenditures, to cope with the financial burden [25, 26]. It may also be worthwhile for the government to revisit the current financial assistance and the co-payment mechanism under certain funds, such as the Samaritan Fund and the Community Care Fund medical assistance programs [27], to address the financial hardship faced by these patients.

The convergent validity of the COST-FACIT-v2 (TC) was supported by its correlation with HRQoL and psychological distress. Consistent with the results of a previous study and review [5, 8], psychological distress showed a positive association with financial toxicity. The presence of correlation showed the instrument’s ability to capture the participants’ psychological characteristics. The findings also imply that to implement appropriate care and follow-up in a timely manner, health care providers should regularly assess cancer patients’ psychological distress from financial toxicity. In addition, a moderate negative correlation was observed between HRQoL and financial toxicity. Cancer patients may adjust their financial needs by reducing their expenditures for basic needs, luxuries and some health-related decisions. Although these adjustments may save money for future treatment expenditures, they may affect their physical and emotional well-being, thereby decreasing HRQoL [25].

The known-group validity was supported by the significant difference obtained in the overall COST-FACIT-v2 (TC) mean score between various income groups and the frequency of using health care services. In this study, the groups with higher household monthly income and those who used fewer health care services had significantly lower overall COST-FACIT-v2 (TC) mean scores, which implies that these patients exhibited more serious financial toxicity. These findings were inconsistent with those obtained in other countries in which co-pay systems are used to reimburse medical costs [8, 28]. This discrepancy could be related to the local provision and choice of available treatment. In Hong Kong, the use of surgery, radiotherapy and chemotherapy in public hospitals is heavily subsidized, and patients need only pay the medical fees that cover both in-patient and follow-up attendance. However, some anti-cancer drugs, targeted therapies and immunotherapy are not subsidized by the government, so patients must make financial arrangements themselves if they opt for those treatment methods [28]. Although patients who cannot afford the medication costs may apply for subsidies from funding bodies such as the Samaritan Fund and the Community Care Fund, not all patients meet the eligibility criteria for such schemes [27]. Hence, further studies may be necessary to further examine the association between treatment methods, income level and the use of health care services.

### Implications for clinical practice

The validated COST-FACIT-v2 (TC) may be a desirable tool in providing rapid assessment of cancer patients’ level of financial toxicity. Its good reliability and validity suggest comparability of findings from Hong Kong and communities that use traditional Chinese or have a publicly funded health care system. More importantly, the COST-FACIT-v2 (TC) generates important information to health care providers for discussion of treatment plans with patients and helps them estimate the potential costs associated with treatment and care. With such discussions, patients can be referred to appropriate personnel, such as medical social workers or financial counsellors, who can assist them with financial planning and application for or exploration of financial assistance programs that help alleviate patients’ financial burden.

### Limitations and recommendations

Despite the study’s desirable findings, some limitations must be acknowledged. The samples show that most recruited patients had stage IV disease, and only 25% had stage I or II disease. It was possible that patients with stage I and II disease may have had less interest in participation in the study because they may have needed only a single treatment that did not incur high medication costs. Meanwhile, because this study was conducted in public hospitals in which cancer treatments are subsidized by the government, the findings may not be generalizable to other settings, such as private hospitals. As a result, further studies in private settings with larger samples of Chinese patients are warranted. Furthermore, the currently reported one- and two-factor structures of the COST-FACIT in the literature were not satisfactorily fitted by our data, the factorial validity of the COST-FACIT-v2 (TC) has not yet been established in our study population. Further studies are warranted to examine the factorial validity of the scale.

## Conclusions

The traditional Chinese version of COST-FACIT-v2 is a valid and reliable instrument that helps health care providers screen for and assess cancer patients’ levels of financial toxicity.

## Data Availability

The data that support the findings of this study are available upon request, but restrictions apply to the availability of these data.

## Abbreviations

A&E: Accident and Emergency Department
CFA: Confirmatory factor analysis
COST-FACIT: COmprehensive Score for financial Toxicity
COST-FACIT-v2: COmprehensive Score for financial Toxicity (Version 2)
COST-FACIT-v2 (TC): COmprehensive Score for financial Toxicity (Version 2)—traditional Chinese
CVI: Content validity index
EFA: Exploratory factor analysis
FACIT: Functional Assessment of Chronic Illness Therapy
FACT-G: Functional Assessment of Cancer Therapy-General
HRQoL: Health-related quality of life
ICC: Intraclass correlation coefficient
I-CVI: Item-content validity index
NNFT: Non-normed fit index
RMSEA: Root mean square error of approximation
S-CVI: Scale-content validity index
SD: Standard deviation
